# Health care policy and market reforms in Japan

**DOI:** 10.1186/1472-6963-14-S2-P58

**Published:** 2014-07-07

**Authors:** Manami Hori

**Affiliations:** 1 Tokai University, Kanagawa, Japan

## 

Since health care markets are usually dictated by domestic and local policies, health care services in Japan are not so much affected by globalization at present. But in some countries, globalization has a great influence on their health care markets as medical tourism is becoming popular around the world. For example, some countries especially developing ones, can attract customers from developed countries by offering high quality health care at a cost lower than in their home country.

The Japanese government has recently acknowledged that health and medical care will certainly form a huge global market in the future. On the other hand, health care services have been under constant pressure to reduce costs for sustainability of social health care insurance. This study discusses how the policy to advance health care markets and that for sustainable social health care insurance can be made compatible in Japan and also argues the risk of policy failures.

